# Genetic Variants Associated with Chronic Obstructive Pulmonary Disease Risk: Cumulative Epidemiological Evidence from Meta-Analyses and Genome-Wide Association Studies

**DOI:** 10.1155/2022/3982335

**Published:** 2022-06-09

**Authors:** Caiyang Liu, Ran Ran, Xiaoliang Li, Gaohua Liu, Xiaoyang Xie, Ji Li

**Affiliations:** ^1^Department of Cardiothoracic Surgery, The First People's Hospital of Neijiang, Neijiang 641000, Sichuan, China; ^2^Breast Surgery Center of Sichuan Cancer Hospital, Chengdu 610041, China

## Abstract

**Background:**

Last two decades, many association studies on genetic variants and chronic obstructive pulmonary disease (COPD) risk have been published. But results from different studies are inconsistent. Therefore, we performed this article to systematically evaluate results from previous meta-analyses and genome-wide association studies (GWASs). *Material and Methods*. Firstly, we retrieved meta-analyses in PubMed, Embase, and China National Knowledge Infrastructure and GWASs in PubMed and GWAS catalog on or before April 7th, 2022. Then, data were extracted and screened. Finally, two main methods—Venice criteria and false-positive report probability test—were used to evaluate significant associations.

**Results:**

As a result, eighty-eight meta-analyses and 5 GWASs were deemed eligible for inclusion. Fifty variants in 26 genes obtained from meta-analyses were significantly associated with COPD risk. Cumulative epidemiological evidence of an association was graded as strong for 10 variants in 8 genes (*GSTM1*, *CHRNA*, *ADAM33*, *SP-D*, *TNF*-*α*, *VDBP*, *HMOX1*, and *HHIP*), moderate for 6 variants in 5 genes (*PI*, *GSTM1*, *ADAM33*, *TNF-α*, and *VDBP*), and weak for 40 variants in 23 genes. Five variants in 4 genes showed convincing evidence of no association with COPD risk in meta-analyses. Additionally, 29 SNPs identified in GWASs were proved to be noteworthy based on the FPRP test.

**Conclusion:**

In summary, more than half (52.38%) of genetic variants reported in previous meta-analyses showed no association with COPD risk. However, 13 variants in 9 genes had moderate to strong evidence for an association. This article can serve as a useful reference for further studies.

## 1. Background

Chronic obstructive pulmonary disease (COPD) is a chronic inflammatory disease with progressive limitation of airflow [1]. According to the latest WHO prediction that COPD will become the third leading cause of death worldwide by 2030, COPD remains a huge health threat and high burden on health care resources to human beings [2]. Smoking and air pollution are widely believed to be the main risk factors of COPD, but only 15–20% of smokers develop this pathology and many COPD cases cannot be explained by environmental risk factors alone [3, 4], suggesting that other factors such as genetic variations may contribute to the development of COPD as well [5]. Moreover, different clinical presentation and severity of COPD between different races and the familial clustering pattern can be seen in COPD cases, indicating substantial evidence of genetic variations to COPD [6, 7]. As early as 2009, Smolonska et al. [8] conducted meta-analyses about 20 polymorphisms in 12 genes and demonstrated that three polymorphisms in *TGF-β1* (rs2241712, rs1982073, and rs6957) were significant with COPD risk in the “diverse populations.” Another three polymorphisms were reported significantly only in Asians (*IL1RN* rs2234663, *TNF-α* rs1800629, and *GSTP1* rs1695). One year later, Castaldi et al. [9] added three more loci (*GSTM1* null variant, *TGF-β1* rs1800470, and *SOD3* rs1799896) related to COPD risk. Follow-up studies reported some other polymorphisms that mainly focused on *IL, MMP, ADRB2, CHRNA, ADAM33, VDBP, SP-A/B/D,* and *COX2*.

Last two decades, a surge of studies investigating genetic polymorphisms and COPD risk have been published, including genome-wide association studies (GWASs), candidate-gene association studies, and meta-analyses. Covering the whole genome, GWASs detect millions of single nucleotide polymorphisms (SNPs) and are a powerful and efficient tool for identifying the association between genetic variants and complex diseases [10]. On the contrary, most of the candidate-gene association studies are underpowered to detect moderate-sized genetic effects. In addition, systematically integrating data from individual studies, meta-analyses have an advantage of developing a single conclusion with greater statistical power [11]. Despite the prominence and growing number of results from GWASs, candidate-gene association studies are still one of the main methods to identify common COPD susceptibility alleles. Some of these associations reported in candidate-gene association studies may be true associations; however, inconsistent results from different studies suggest the possibility of false-positive associations. Even results from meta-analyses cannot be replicated in follow-up studies, indicating that these associations lack robustness. Guidelines for assessing the cumulative epidemiological evidence of genetic associations, known as the Venice criteria, were proposed by the Human Genome Epidemiology Network (HuGENet) in 2008 [12]. These guidelines help evaluate gene-disease associations [13]. However, as far as we know, there is still no such integrative and updated evaluation for all the reported polymorphisms associated with COPD in current literature.

Therefore, we collected evidence from meta-analyses and GWASs to conduct an integrative assessment of the gene-COPD associations based on the Venice criteria and false-positive report probability (FPRP) test, thus providing a synopsis of our current understanding of the genetic basis of COPD risk.

## 2. Materials and Methods

### 2.1. Study Eligibility and Literature Search

A systematic strategy was used to identify all relevant publications. Firstly, we searched PubMed, Embase, and China National Knowledge Infrastructure (CNKI) using the terms “COPD or chronic obstructive pulmonary disease,” “genetic or genetic association or single nucleotide polymorphism or SNP or polymorphism or genotype or variant or mutation or susceptibility,” and “meta-analysis or systematic review or literature review” for meta-analyses. We searched PubMed using the terms “COPD or chronic obstructive pulmonary disease” and “GWAS or genome-wide association study” as well as checking the GWAS catalog for GWASs on or before April 7th, 2022. Secondly, after manually screening the title and abstract, all references cited in relevant studies were also reviewed to identify additional studies. Inclusion criteria for meta-analyses were as follows: data were published in a peer-reviewed journal; studies used a cross-sectional, case-control, or cohort design; the association was about the etiology of COPD; and necessary information was provided for the FPRP test or/and Venice criteria. GWASs were eligible for inclusion if they met the following criteria: the association was directly about the etiology of COPD; *P* value <5 × 10^−8^; and both discovery and replication phases were reported. We removed duplicated and unrelated articles by screening the title and abstract or reading the full-text if necessary. Literature search and studies screening were done by Liu and Ran together. Any disagreement was resolved by consensus.

### 2.2. Data Extraction

Two reviewers, Liu and Ran, extracted data separately and then exchanged and cross-checked. Any disagreement was resolved by consensus. We collected the following data from meta-analyses: PMID, gene name, genetic variant, first author, publication year, comparison model, ethnicity, OR and 95% CI, adjustment for smoking (Yes/No), *I*^2^, the number of studies, cases and controls, minor allele frequency (MAF) in controls, *P* value of Egger's test and *P* value of the *Q* test, and the number of test alleles or genotypes. We collected the following data from GWASs: PMID, gene name, genetic variant, first author, publication year, risk allele, ethnicity, OR and 95% CI, risk allele frequency, *P* value, and the number of cases and controls. Caucasian and Asian were the two major ethnicities reported. We defined ethnicity as “diverse populations” if a combination of two or more ethnicities were reported. We extracted significant results of subgroup analysis stratified by ethnicity. Cigarette smoking is the major environmental risk factor for COPD and may influence the distribution of genetic polymorphisms [14]; therefore, results after adjusted for smoking were extracted as well. Except several variants (e.g., *PIMZ* and *TGF-β1* rs2241718), all other were investigated exclusively by one meta-analysis, most of the variants were widely investigated. We extracted data from them all, but only one meta-analysis was chosen for further assessment considering publication year, comparison model, sample size, between-study heterogeneity, and study design altogether. Because there was a considerable overlap among these studies, to avoid the variant nomenclature confusion from different articles, we used the uniform identifiers (“rs” number) of variants in the dbSNP. For the variants without any “rs” number, we used the common nomenclature (e.g., *GSTT1* null/present and *SERPINA3* Ala9Thr). Associations were considered statistically significant if the reported *P* value was less than 0.05 or if the 95% CI excluded 1.0. Most of the meta-analyses provided multiple results under different genetic models; in order to avoid selection bias, we used the allelic model as the unified model. When data under allelic model were not available, then other models were used. Statistical analyses were performed with Stata, version 12.0, using the original data provided in meta-analyses if there was no sufficient information for the Venice criteria.

### 2.3. Evaluation of Cumulative Evidence

For statistically significant associations from meta-analyses, we applied the Venice criteria to grade the credibility of cumulative epidemiological evidence. Three aspects were assigned to the Venice criteria: the amount of evidence, replication of association, and protection from bias. For each of the three aspects, three levels (A, B, or C) were assigned, based on which credibility was defined as strong, moderate, or weak. Amount of evidence was determined by the sum of test alleles or genotypes among cases and controls: A (*n* > 1000), B (100 < *n* < 1000), and C (*n* < 100). We did not apply this criterion for rare variants with frequency less than 1% since an A grade was unlikely to obtain [15]. The replication of association was determined depending on between-study heterogeneity: A (*I*^2^ < 25%), B (25% < *I*^2^ < 50%), and C (*I*^2^ > 50%). Protection from bias should take various potential sources of bias into consideration. A grade of A was assigned when there was no demonstrable bias or the bias would unlikely invalidate the association. A grade of B was assigned when there was no sufficient information for identifying evidence although there was no obvious bias. A grade of C was assigned when the bias was evident or/and was likely to explain the presence of association. Furthermore, if any of the following situations occurred, a grade of C was assigned: the sensitivity analysis indicated that the significant results can be substantially changed; the magnitude of the association was low (e.g., OR < 1.15 and OR > 0.87 for risk effect and protective effect, respectively) unless the association had been replicated prospectively by GWASs or several studies with no evidence of publication bias; and obvious publication bias (Egger's test *P* value <0.05). Cumulative epidemiological evidence was categorized as strong if all grades were A and weak if any grade was C. All of the rest combinations were categorized as moderate. If the data were insufficient, then associations were not evaluated (*n* = 6).

An approach developed by Wacholder et al. [16] was also used to calculate the FPRP for all the significant associations. The FPRP was determined by three parameters: the observed *P* value, the statistical power, and the prior probability. We used the prior probability of 0.01 and preset the FPRP noteworthiness value at 0.2 in our study. FPRP values and statistical power were calculated by the excel spreadsheet offered by Wacholder et al. We considered the association noteworthy if the calculated FPRP value was less than 0.2, indicating a true association. FPRP < 0.05, 0.05 < FPRP < 0.2, and FPRP > 0.2 were considered strong, moderate, and weak evidence of true association, respectively. We upgraded cumulative epidemiological evidence from moderate to strong, from weak to moderate, if evidence based on the FPRP was strong. We downgraded cumulative epidemiological evidence from strong to moderate, from moderate to weak, if evidence based on the FPRP was weak [17].

## 3. Results

### 3.1. Overall Characteristics

**Figure 1:**
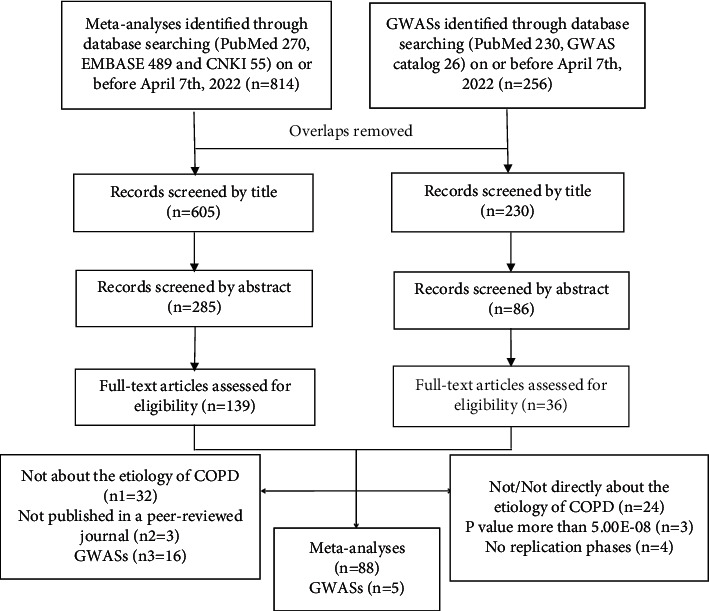
Selection of studies.

A total of 88 meta-analyses met the eligibility criteria (Figure 1), reporting 86 variants in 40 genes. One hundred and fourteen associations (66 significant associations and 48 nonsignificant associations) were addressed between these variants and the risk of COPD. Except 9 associations, results were obtained based on at least three original studies. The included meta-analyses had a mean of 1822 cases (range 120–10466) and 4848 controls (range 100–95336). Publication time ranged from 2004 to 2019, and most of them (*n* = 77, 92.8%) were published since 2010. More detailed information was presented in the tables.

A total of 36 GWASs were included for full-text assessment (Figure 1), but most of them were excluded for not/not directly about the etiology of COPD. The *P* value of 3 GWASs did not meet the threshold of 5 × 10^−8^ [18–20], and in another 4 GWASs there was a lack of replication phases [21–24]. At last, 5 GWASs met the criteria for inclusion and reported 32 SNPs in or near 25 genes [25–29].

### 3.2. Significant Associations in Meta-Analyses

As mentioned above, 66 significant associations were identified, including 38 associations obtained from the main meta-analyses and 28 associations obtained from the subgroup meta-analyses [30–57]. These associations involved 50 variants in 26 genes. In the main meta-analyses, 43 variants reported ORs higher than 1.0 with a mean value of 1.61 (range 1.14–3.26, median 1.43), while protective effect was found for the other 7 variants with a mean value of 0.70 (range 0.51–0.86, median 0.76). In the subgroup meta-analyses, 16 variants reported ORs higher than 1.0 with a mean value of 1.66 (range 1.14–2.64, median 1.53), while protective effect was found for the other 7 variants, with a mean value of 0.65 (range 0.46–0.79, median 0.63). The average number of included studies in the main meta-analyses was 9, while the median was 6 (range 1–38). The average number of total sample size was 7818, while the median number was 4168 (range 220–49520). Most of the meta-analyses contained diverse ethnicities, while only three reported one specific ethnicity, including one for Caucasians and two for Asians.

The Venice criteria and FPRP test were used to evaluate the epidemiological credibility of the 66 significant associations (Table 1). Firstly, the Venice criteria was applied, as a result grade A was given to 36, 21, and 53 associations for amount of evidence, replication of association, and protection from bias, respectively. Grade B was given to 25, 14, and 2 associations for these three aspects, respectively. Grade C was given to 3, 30, and 5 associations for these three aspects, respectively. It is worth nothing that the amount of evidence was not applied for one variant, *PISZ*, because of its low frequency (0.12%). As a result, 10, 17, and 33 associations were categorized as strong, moderate, and weak, respectively. We did not evaluate the other 6 associations because of the insufficient information. Secondly, FPRP was applied to test the evidence of true association. As a result, 17, 7, and 42 associations were categorized as strong, moderate, and weak, respectively. Finally, we upgraded cumulative evidence from moderate to strong for *GSTM1* null/present in Caucasians, *CHRNA* rs1051730 in non-Asians, *ADAM33* rs612709, *SP-D* rs721917 in Asians, *TNF*-*α* rs1800629 in Asians, and *HMOX1* L allele in Asians and from weak to moderate for *GSTM1* null/present, *GSTM1* null/present in Asians, and *TNF*-*α* rs1800629. We downgraded cumulative evidence from strong to moderate for *PIMS*, *ADAM33* rs3918396, and *ADAM33* rs3918396 after adjusted for smoking and from moderate to weak for *IL-13* rs1800925 in Caucasians, *ADAM33* rs2280091 and rs511898, *CYP1A1* rs4646903, *TNF-α* rs1800630, *VDBP* 1S, *COX2* rs20417, *IREB2* rs2568494, and *ADRB2* rs1042714 in Asians. Altogether, cumulative epidemiological evidence of 13 associations involved 10 variants in 8 genes were considered to be strong, including *GSTM1* null/present in Caucasians, *CHRNA* rs16969968, rs8034191, and rs1051730, *CHRNA* rs1051730 in non-Asians, *ADAM33* rs612709, *ADAM33* rs612709 in Caucasians, *SP-D* rs721917, *SP-D* rs721917 in Asians, *TNF*-*α* rs1800629 in Asians, *VDBP* 1F, *HHIP* rs13118928, and *HMOX1* L allele in Asians. Eight associations involved 6 variants in 5 genes (*PI*, *GSTM1*, *ADAM33*, *TNF*-*α*, and *VDBP*) and 39 associations involved 40 variants in 23 genes were considered to be moderate and weak evidence, respectively.

### 3.3. Significant Associations in GWASs

Thirty-two SNPs were reported in GWASs (Table 2), among which 25 SNPs showed an association with increased COPD risk with a mean OR value of 1.17 (range 1.08–1.36, median 1.15), while protective effect was found for 4 SNPs with a mean OR value of 0.73 (range 0.73–0.74, median 0.73). There were no sufficient data in GWASs for the rest 3 SNPs. Unsurprisingly, based on the FPRP test, all the 29 SNPs were proved to be noteworthy. Given that the Venice criteria was not applicable to GWASs, we did not further evaluate these results. Additionally, we extracted data of another 77 SNPs from the two GWASs excluded for lacking of replication phases. Because the study of Sakornsakolpat et al. [21] is one of the largest COPD GWASs to date (Supplementary Table 1), these data might provide an overview of known and well-established COPD SNPs from GWASs.

### 3.4. Nonsignificant Associations in Meta-Analyses

Forty-eight statistically nonsignificant associations were reported for 55 variants in 27 genes (Supplementary Table 2) [8, 9, 34–37, 39, 42, 44, 45, 47, 54, 55, 58–69]. These meta-analyses included a mean of 6 studies (range 1–15) and 5587 participants (range 220–100777). Except 48 statistically nonsignificant associations, 9 significant associations were identified in the stratified analysis by ethnicity [8, 36, 42, 54, 55, 59, 65, 70]. Only 4 associations met the following criteria for further assessment: genetic model must be an allelic model, and significant association was congruously confirmed across studies. However, all of them were graded as weak evidence of association with COPD risk. Additionally, 5 variants (*TGF*-*β1* rs1800469, *MMP-1* rs1799750, *SERPINE2* rs3795879, and *CHRNA* rs578776 and rs588765) in 4 genes showed convincing evidence of no association with COPD risk in meta-analyses that included a minimum of 2400 cases and 3000 controls [34, 62, 67, 68].

### 3.5. Inconsistency among Meta-Analyses

Controversial results were reported for 23 variants in the meta-analyses (Supplementary Table 3) [8, 9, 32, 34–36, 38, 41, 42, 44–51, 57, 58, 61–64, 69–97]. Considering all of the factors we mentioned in “Data Extraction” comprehensively, 15 variants were deemed to have a significant association with COPD risk: *TNF*-*α* rs1800629 and rs1800630, *GSTT1* null/present, *MMP9* rs3918242, *EPHX1* rs1051740 and rs2234922, *IL-13* rs1800925, *IL-6* rs1800796, *ADAM33* rs2280091, rs511898, and rs612709, *SP-D* rs721917, *VDBP* 1F and 1S, and *HHIP* rs13118928. Eight variants were deemed to have a nonsignificant association with COPD risk: *TNF*-*α* rs1800610, *TGF*-*β1* rs2241712, rs1982073, and rs6957, *IL1β* rs1143627, *SOD3* rs1799895, *ADAM33* rs528557, and *VDBP* GC-2. Still, results of seven variants should be prudently interpreted since studies with the similar sample size yielded opposing results, including *TNF*-*α* rs1800630, *TGF*-*β1* rs2241712, *ADAM33* rs2280091, rs612709, and rs511898, *IL-6* rs1800796, and *VDBP* 1S.

## 4. Discussion

As far as we know, this study is the only one which aims to give a comprehensive assessment on all the reported genetic variants and COPD susceptibility with systematic methods. We retrieved relevant meta-analyses and GWASs, extracting useful data for further evaluation. The Venice criteria and FPRP test were the two major tools. As a result, 10 variants in 8 genes were graded as strong evidence of association with COPD risk, including: *GSTM1* null/present, *CHRNA* rs16969968, rs8034191, and rs1051730, *ADAM33* rs612709, *SP-D* rs721917, *TNF*-*α* rs1800629, *VDBP* 1F, *HMOX1* L allele, and *HHIP* rs13118928. Six variants in 5 genes and 40 variants in 23 genes were graded as moderate and weak evidence, respectively. In addition, 29 SNPs identified in GWASs were proved to be noteworthy. There were overlaps between the two groups of SNP. Four SNPs (*CHRNA* rs1051730 and rs8034191, *SP-D* rs721917, and *FAM13A* rs7671167) reported in GWASs were also investigated by meta-analyses. Except *FAM13A* rs7671167, all the other three SNPs were graded as strong evidence, suggesting that methods we used do have the ability to pick out potential SNPs as long as a high-quality meta-analysis was included.

GSTM is a kind of the glutathione S-transferase (GST) cytoplasmic enzymes that metabolize various toxic substances [98]. *GSTM1*, located on chromosome 1p13.3, is highly expressed in the lung tissue. *GSTM1* homozygous deletion leads to the absence of protein expression and production, preventing the detoxification. Previous studies have proven that the null genotype of *GSTM1* was related to the increased risk of inflammatory lung diseases, thus taking an important part in COPD development [99]. Our study demonstrated that *GSTM1* null genotype showed strong cumulative evidence for an association with COPD risk in Caucasians and moderate cumulative evidence was found in “diverse populations” and Asians. In addition, highly consistent positive results were reported in recent meta-analyses [8, 9, 32, 78, 79]. The association between *GSTM1* null genotype and COPD risk was well established.

*CHRNA3/5*, located on chromosome 15q25, encodes the subunits of alpha-nicotinic acetylcholine receptor (nAChR). Gwilt et al. [100] reported that nAChR might play a role in modifying the inflammatory response to smoking. Moreover, variants in *CHRNA3/5* were involved in altering mRNA levels of *CHRNA5* in the lung tissue and influencing receptor response to nicotine agonists [101, 102]. Therefore, we can draw a conclusion that these variants may contribute to the development of COPD. Four variants (rs16969968, rs8034191, rs1051730, and rs6495309) in *CHRNA3/5* were reported significantly in our study. Except rs6495309, all of them showed strong cumulative evidence for an association with COPD risk. Interestingly, two variants were also identified as susceptibility loci for COPD by GWASs [25]. However, these strong associations were limited to non-Asians because distribution of the minor allele varies extensively between different ethnicities. Hence, we recommend additional studies to confirm the association between these polymorphisms and COPD risk in Asians.

*ADAM33*, located on chromosome 20p13, is a member of the “a disintegrin and metalloprotease” (*ADAM*) family and is intricately related to airway hyper-responsiveness and obstruction [103]. Rs612709 in *ADAM33* showed protective effect and was rated as strong evidence of association with COPD in both “diverse populations” (OR = 0.60, 95% CI = 0.52−0.68) and Caucasians (OR = 0.64, 95% CI = 0.53−0.77). Another meta-analysis with the similar publication year and data source yet yielded an opposing result (OR = 1.46, 95% CI = 1.14−1.87) [41]. A cognitive error in the minor allele may lead to this conflict (rs612709G>A).

*SP-D*, also known as *SFTPD*, is a kind of alveolar surfactant-associated protein (SP) and plays a prominent role in maintenance of lung function and immune regulation [104]. Our study revealed strong evidence for an association between the T allele of rs721917 and COPD risk in both “diverse populations” and Asians based on a total of 1111 and 934 subjects, respectively. Although sample size is relatively small, the high frequency (>40%) of the T allele makes it possible to achieve sufficient statistical power. However, no significant association was reported in Caucasians because data from Caucasians were scarce. Hence, further studies that focus on Caucasians are warranted.

*TNF*-*α* is a pivotal cytokine in inflammation of the lung [105]. Rs1800629 in *TNF*-*α* contains a G to A variation, carriers of the A allele are more likely to activate the *TNF*-*α* promoter region and lead to overexpression of *TNF*-*α* [106]. Highly consistent positive results of association between rs1800629 A allele and COPD risk in Asians but not in Caucasians were reported [8, 46, 71–75]. Our study also graded rs1800629 as having strong evidence of association with COPD risk in Asians. Nevertheless, no correlation was found between rs1800629 and COPD risk after adjustment for smoking (OR = 1.13, 95% CI = 0.95−1.35). It seems that other factors may contribute to the risk of COPD in smokers. Expanding studies with a sufficient number of smokers are needed.

Heme oxygenase plays an important role in resisting damage caused by oxidative stress. *HMOX1*, belongs to the heme oxygenase isoforms, was reported to have the ability of protecting against airway inflammation and emphysema [107, 108]. The L allele (long GT repeat sequence) of *HMOX1* was rated as strong evidence of association with 2.23-fold increased risk of COPD based on 923 subjects in Asians. The sample size was relatively small, and the frequency of the L allele was low (9.29%). Additionally, only one meta-analysis was retrieved in our study. Although we used the Venice criteria and FPRP test to evaluate the credibility of an association, a firm conclusion should not be drawn until studies with more subjects can be included in the meta-analysis.

*HHIP* gene, located on chromosome 4q31, encodes an inhibitory protein for sonic hedgehog. The hedgehog is known to be essential for branching morphogenesis in the developing lung [109]. Zhou et al. [110] demonstrated that *HHIP* expression at both mRNA and protein levels is reduced in COPD lung tissues. These studies showed that *HHIP* plays an important role in maintaining the normal lung function. *HHIP* rs13118928 was firstly reported by Pillai et al. [25] in GWASs but did not reach genome-wide significance levels. However, follow-up studies [98, 111] including ours have proved the association.

*VDBP*, located on chromosome 4q12-q13, is also known as *GC* and participates in binding substantial quantities of 25-hydroxyvitamin D and vitamin D [111]. Two mutations (C⟶T and G⟶A) of SNPs (rs4588 and rs7041) in *VDBP* result in 3 common isoforms (*GC1S*, *GC1F*, and *GC2*) and different protein products. We found strong and moderate evidence of association between *GC1F* and the risk of COPD in “diverse populations” and Asians, respectively. Among 8 meta-analyses we retrieved, only one meta-analysis reported a significant association with decreased risk of COPD in Caucasians [94]. The association between polymorphisms and COPD risk varied between ethnicities. Reasons may be summarized as follows: different genetic backgrounds, different degrees of environmental exposure, and small sample size results in poor statistical power.

Six variants in 5 genes and 40 variants in 23 genes showed moderate and weak evidence of association with COPD risk in our study, respectively. Among which, two variants (*PIMS* and *ADAM33* rs3918396) and eight variants (*IL-13* rs1800925, *ADAM33* rs2280091, *CYP1A1* rs4646903, *TNF*-*α* rs1800630, *VDBP* 1S, *COX2* rs20417, *IREB2* rs2568494, and *ADRB2* rs1042714) were downgraded from strong to moderate and moderate to weak due to high FPRP values (>0.2), respectively. As we mentioned in “Materials and Methods,” three parameters may influence the calculated FPRP values. We used the prior probability of 0.01 for FPRP calculations; however, the calculated FPRP values might be smaller if a softer prior probability was used (e.g., 0.05) [17]. Therefore, we recommend further investigations on these variants since significant associations may have been excluded.

There was one more variant worth noting, *FAM13A* rs7671167; this showed weak evidence of association with COPD susceptibility because of the strong heterogeneity between studies. But it was identified by GWASs [27]. Results from GWASs, however, are more statistically significant and convincing. The Venice criteria, undoubtedly, is a useful tool to evaluate the cumulative epidemiological evidence of associations between SNPs and diseases. But sometimes omissions might occur because “weak evidence” was identified as long as a “C” was assigned to any of the three aspects. Results would be more credible if a more high-quality meta-analysis was included or different weights were assigned to the three aspects included in the Venice criteria.

Forty-eight statistically nonsignificant associations were reported for 55 variants in 27 genes. Five variants (*TGF-β1* rs1800469, *MMP-1* rs1799750, *SERPINE2* rs3795879, and *CHRNA* rs578776 and rs588765) showed no association with COPD risk in meta-analyses including a minimum of 2400 patients and 3000 controls. The MAFs of these variants ranged from 20% to 50%. In other words, these meta-analyses provided greater than 88% power to detect an OR of 1.15 under different genetic models for these variants. In addition, no inconsistency was found among meta-analyses investigating these variants; therefore, we can safely draw a conclusion that these variants are not associated with the risk of COPD. Further studies are unlikely to yield significant results.

Limitations of our study must be addressed. Scopus and Web of Science were not retrieved. Non-English literature except literature in Chinese and gray literature were not included. Although a systematic literature search was done, and some articles were overlooked. Only one meta-analysis was chosen for further evaluation, which might introduce bias to some extent. The latest candidate-gene association studies might be missed. We extracted results from subgroup analyses to address the heterogeneity, but heterogeneity does exist. High heterogeneity influenced the evaluation directly.

## 5. Conclusions

In summary, combining Venice criteria and FPRP test, cumulative epidemiological evidence of significant associations between genetic variants and COPD risk was evaluated. As a result, 13 variants showed moderate to strong evidence. Our study can provide not only reliable evidence but also helpful clues for further investigations.

## Figures and Tables

**Table 1:** Statistically significant variants from meta-analyses, false-positive report probabilities (FPRP), and cumulative epidemiological evidence.

Gene	Variants	Year	Comparison	Ethnicity	OR (95% CI)	Adjustment for smoking	Publication bias/heterogeneity^∗∗^	*I* ^2^ (%)	No. of studies	Cases/control	Maf (%)^†^	Number of test alleles or genotypes	Venice criteria	Power OR of 1.5	FPRP values at prior probability of 0.01	Cumulative epidemiological evidence	Reference
*PI*	PIMZ	2004	PIMZ vs PIMM	Diverse	2.31 (1.60–3.35)	No	0.15/0.002	58.6^*∗*^	16	3653/12340	4.43	822	BCC^‡^	0.011	0.081	Weak	[30]
			PIMZ vs PIMM	Diverse	1.61 (0.92–2.81)	Yes	No/0.15	NA	5	1600/7645	—	—	—	—	—	—	[30]
*PI*	PISZ	2005	PISZ vs PIMM	Diverse	3.26 (1.24–8.57)	No	0.98/0.23	27.2^*∗*^	6	2404/8999	0.12	42	NBC^‡^	0.058	0.966	Weak	[31]
*PI*	PIMS	2005	PIMS vs PIMM	Diverse	1.19 (1.02–1.38)	No	1.0/0.54	0.0^*∗*^	17	3630/13030	7.26	1169	AAA	0.999	0.679	Moderate	[31]
			PIMS vs PIMM	Diverse	1.02 (0.81–1.28)	Yes	NA/NA	NA	5	1526/7859	—	—	—	—	—	—	[31]
*GSTM1*	Null/present	2018	Null vs present	Diverse	1.52 (1.31–1.77)	No	No/<0.00001	64.0	37	4669/4914	46.99	5018	ACA	0.432	0.000	Moderate	[32]
			Null vs present	Asian	1.59 (1.29–1.96)	No	No/<0.0001	64.0	23	2374/2630	44.79	2531	ACA	0.293	0.005	Moderate	[32]
			Null vs present	Caucasian	1.27 (1.11–1.44)	No	No/0.03	49.0	11	1881/1938	49.85	2045	ABA	0.995	0.019	Strong	[32]
*GSTT1*	Null/present	2018	Null vs present	Diverse	1.28 (1.09–1.50)	No	No/0.001	51.0	29	3273/3578	32.36	2338	ACA	0.975	0.188	Weak	[32]
			Null vs present	Asian	1.30 (1.05–1.61)	No	No/0.002	57.0	18	2025/2253	39.61	1697	ACA	0.905	0.639	Weak	[32]
*TGF-b1*	rs1800470	2011	C vs T	Diverse	0.82 (0.70–0.96)	No	0.326/0.01	57.6	10	1507/2542	43.14	3533	ACA	0.995	0.575	Weak	[33]
			C vs T	Caucasian	0.77 (0.60–0.98)	No	No/0.02	65.9	5	840/1788	41.11	2061	ACA	0.879	0.791	Weak	[33]
*TGF-β1*	rs1982073※	2017	C vs T	Caucasian	0.79 (0.64–0.99)	No	No/0.006	75.7	4	1656/2564	40.72	3348	ACA	0.930	0.812	Weak	[34]
*IL1RN*	rs2234663	2019	L vs 2	Diverse	0.56 (0.39–0.80)	No	0.02/0.25	30.3	5	322/365	10.11	171	BBC^‡^	0.169	0.458	Weak	[35]
*IL-6*	rs1800796	2019	CC + GC vs GG	Diverse	2.23 (1.46–3.40)	No	0.05/0.38	19.3	3	574/862	26.33	511	BAC^‡^	0.033	0.370	Weak	[35]
*IL-13*	rs1800925	2017	C vs T	Diverse	1.57 (1.21–2.04)	No	No/0.015	57.9	9	1477/891	16.27	963	BCA	0.366	0.166	Weak	[36]
			C vs T	Asian	1.88 (1.23–2.87)	No	0.268^*∗*^/0.016	67.1	5	555/544	14.71	425	BCA	0.148	0.698	Weak	[36]
			C vs T	Caucasian	1.30 (1.01–1.67)	No	0.644^*∗*^/0.314	15.6	4	922/347	18.73	538	BAA	0.869	0.820	Weak	[36]
*IL-27*	rs153109	2016	G vs A	Asian	0.51 (0.34–0.74)	No	NA/NA	NA	1	120/100	45.50	NA	XXX	0.079	0.329	NA	[37]
*MMP-9*	rs3918242	2017	T vs C	Diverse	1.32 (1.02–1.71)	No	0.12/<0.001	77.0	13	2295/2539	11.17	1408	ACA	0.833	0.808	Weak	[38]
			CT + TT vs CC	Asian	1.80 (1.04–3.11)	No	No/<0.001	85.0	8	1101/1147	14.34	645	BCA	0.257	0.931	Weak	[38]
*MMP-12*	rs652438	2014	G vs A	Diverse	1.62 (1.08–2.42)	No	>0.05/0.070	57.5	4	710/1652	10.57	328	BCA	0.354	0.838	Weak	[39]
*CHRNA*	rs16969968	2014	C vs T	Diverse	1.27 (1.17–1.39)	No	No/0.655	0.0	3	1996/6463	29.42	4979	AAA	1.000	0.000	Strong	[40]
*CHRNA*	rs1051730	2014	T vs C	Diverse	1.14 (1.10–1.18)	No	No/0.318	14.0	9	10466/39054	30.80	30264	AAA^§^	1.000	0.000	Strong	[40]
			T vs C	Non-Asian	1.14 (1.10–1.18)	No	No/0.124	42.2	6	8620/37099	32.32	30088	ABA^§^	1.000	0.000	Strong	[40]
*CHRNA*	rs8034191	2014	C vs T	Diverse	1.29 (1.18–1.41)	No	No/0.644	0.0	5	2652/2565	24.09	2933	AAA	1.000	0.000	Strong	[40]
*CHRNA*	rs6495309	2014	C vs T	Diverse	1.26 (1.09–1.45)	No	Pre/0.109	50.4	4	1977/2131	48.12	3756	ACC	0.993	0.112	Weak	[40]
*ADAM33*	rs3918396	2014	GG + AG vs AA	Diverse	1.42 (1.12–1.79)	No	0.168/0.89	0.0	6	1422/3034	25.61	3806	AAA	0.679	0.304	Moderate	[41]
			GG + AG vs AA	Asian	1.45 (1.14–1.85)	No	NA/0.665^*∗*^	0.0	3	642/630	67.38	645	BAX	0.607	0.313	NA	[41]
			GG + AG vs AA	Diverse	1.45 (1.14–1.84)	Yes	No/0.91	0.0	3	836/848	54.07	1052	AAA	0.610	0.266	Moderate	[41]
*ADAM33*	rs597980	2014	A vs G	Caucasian	1.25 (1.05–1.48)	No	NA/0.158	49.9	2	296/2294	40.87	2148	ABX	0.983	0.492	NA	[42]
*ADAM33*	rs2280091	2014	GG vs AA + AG	Diverse	1.76 (1.27–2.43)	No	0.483/0.348	10.5	8	2112/3952	13.18	197	BAA	0.166	0.262	Weak	[42]
			G vs A	Asian	2.03 (1.40–2.94)	No	0.857/0.006	76.0	4	1130/1135	13.04	798	BCA	0.055	0.245	Weak	[42]
*ADAM33*	rs511898	2019	A vs G	Diverse	1.16 (1.04–1.30)	No	0.726/0.048	44.5	12	2454/4481	33.17	4763	ABA	1.000	0.514	Weak	[51]
*ADAM33*	rs612709	2014	A vs G	Diverse	0.60 (0.52–0.68)	No	0.070/0.107	44.7	6	1341/3359	67.61	5345	ABA	0.050	0.000	Strong	[42]
			A vs G	Asian	0.61 (0.42–0.89)	No	0.256/0.021	74.1	3	767/763	26.54	664	BCA	0.322	0.760	Weak	[42]
			A vs G	Caucasian	0.64 (0.53–0.77)	No	0.324/0.798	0.0	3	574/2596	79.68	4681	AAA	0.333	0.001	Strong	[42]
*ADAM33*	rs2280090※	2014	A vs G	Asian	0.46 (0.33–0.66)	No	0.581/0.021	74.1	3	767/763	32.31	763	BCA	0.022	0.101	Weak	[42]
*CYP1A1*	rs4646903	2015	CC vs TT	Diverse	1.73 (1.18–2.55)	No	No/0.34	11.0	5	532/591	23.43	141	BAA	0.236	0.703	Weak	[43]
			CC vs TT	Asian	1.84 (1.11–3.06)	No	NA/0.65	0.0	2	158/154	35.27	88	CAX	0.216	0.896	NA	[43]
*CYP1A1*	rs1048943	2015	GG vs AA	Diverse	3.23 (1.50–6.93)	No	No/0.16	45.0	3	510/553	9.01	41	CBA	0.024	0.914	Weak	[43]
*SP-A*	Common polymorphisms	2013	W vs M	Diverse	1.53 (1.14–2.05)	No	>0.05/0.043	56.5	3	383/369	23.83	828	BCA	0.447	0.493	Weak	[44]
*SP-D*	rs721917	2019	T vs C	Diverse	1.39 (1.18–1.65)	No	0.375/0.901	0.0	5	561/550	45.36	1106	AAA	0.808	0.020	Strong	[45]
			T vs C	Asian	1.44 (1.20–1.73)	No	0.457/0.978	0.0	4	499/435	43.56	917	BAA	0.669	0.014	Strong	[45]
*SP-A/B/D*	Common polymorphisms	2013	W vs M	Asian	1.43 (1.15–1.78)	No	>0.05/0.001^*∗*^	71.3^*∗*^	4	485/488	27.28	1080	ACA	0.666	0.169	Weak	[44]
*TNF-α*	rs1800629	2016	A vs G	Diverse	1.56 (1.29–1.89)	No	No/0.000	70.8	38	3951/5110	10.96	2326	ACA	0.344	0.002	Moderate	[46]
			A vs G	Asian	2.40 (1.98–2.90)	No	No/0.093	29.8	22	2067/2167	7.63	1011	ABA	0.000	0.000	Strong	[46]
			A vs G	Diverse	1.13 (0.95–1.35)	Yes	No/0.089	34.9	15	1500/1239	—	—	—	—	—	—	[46]
			A vs G	Asian	1.26 (0.69–2.30)	Yes	No/0.063	52.2	6	570/616	—	—	—	—	—	—	[46]
*TNF-α*	rs1800630	2013	A vs C	Diverse	0.76 (0.60–0.97)	No	No/0.158	42.3	4	606/622	16.64	155	BBA	0.854	0.761	Weak	[47]
			A vs C	Asian	0.56 (0.37–0.85)	No	NA/0.640	0.0	2	202/203	16.50	40	CAX	0.206	0.756	NA	[47]
*EPHX1*	rs1051740	2012	CC vs TT	Diverse	1.33 (1.06–1.69)	No	0.158/<0.0001	73.0	25	4808/24888	52.59	5186	ACA	0.837	0.699	Weak	[48]
			CC vs TT	Caucasian	1.61 (1.12–2.31)	No	No/<0.0001	81.0	12	3930/23907	49.91	4343	ACA	0.350	0.733	Weak	[48]
*EPHX1*	rs2234922	2012	Extremely slow vs normal	Diverse	1.77 (1.23–2.55)	No	No/<0.0001	76.0	15	3146/21391	NA	4180	ACA	0.187	0.535	Weak	[48]
			Slow vs normal	Diverse	1.44 (1.13–1.85)	No	No/<0.00001	74.0	15	4248/28597	NA	12488	ACA	0.625	0.407	Weak	[48]
			Extremely slow vs normal	Caucasian	2.64 (1.30–5.38)	No	No/<0.0001	85.0	8	2685/20884	NA	3813	ACA	0.060	0.926	Weak	[48]
			Slow vs normal	Caucasian	1.31 (1.01–1.71)	No	No/0.01	62.0	8	3563/27956	NA	11763	ACA	0.840	0.847	Weak	[48]
*VDBP*	1F	2015	1F vs 1S	Diverse	1.47 (1.22–1.77)	No	No/0.447	0.0	8	809/1407	20.47	1125	AAA	0.584	0.008	Strong	[49]
			1F vs 1S	Asian	1.53 (1.22–1.92)	No	0.133/0.935	0.0	5	510/460	30.84	319	BAA	0.432	0.052	Moderate	[49]
*VDBP*	1S	2015	1S vs non 1S	Diverse	0.86 (0.77–0.96)	No	No/0.12	35.0	11	1410/1595	46.14	2578	ABA	1.000	0.416	Weak	[50]
			1S vs non 1S	Asian	0.76 (0.64–0.89)	No	No/0.23	26.0	7	785/719	31.71	869	BBA	0.948	0.064	Moderate	[50]
*HHIP*	rs13118928	2019	G vs A	Diverse	1.14 (1.08–1.20)	No	No/0.930	0.0	7	5157/9768	36.39	10250	AAA^§^	1.000	0.000	Strong	[57]
*COX2*	rs20417	2014	C vs G	Diverse	1.33 (1.06–1.67)	No	>0.05/0.209	30.2	6	930/719	17.04	597	BBA	0.850	0.621	Weak	[39]
*IREB2*	rs2568494	2016	AA vs GG + AG	Diverse	1.38 (1.09–1.76）	No	No/0.171	40.1	4	2001/2167	22.70	322	BBA	0.749	0.555	Weak	[52]
*HMOX1*	L allele	2017	L vs S + M	Diverse	2.02 (1.31–3.11)	No	0.79/0.08	52.0	5	601/547	8.78	287	BCB	0.088	0.612	Weak	[53]
			L vs S + M	Asian	2.23 (1.68–2.95)	No	No/0.46	0.0	4	471/452	9.29	262	BAB	0.003	0.001	Strong	[53]
*ACE*	I/D※	2018	D vs I	Asian	1.63 (1.17–2.27)	No	No/0.065	54.7	5	380/206	39.86	685	BCA	0.311	0.549	Weak	[54]
*ADRB2*	rs1042714※	2018	G vs C	Asian	1.29 (1.02–1.65)	No	0.970^*∗*^/0.34	10.0	4	613/587	11.46	314	BAA	0.885	0.826	Weak	[55]
*FAM13A*	rs7671167	2017	CC + CT vs TT	Diverse	0.76 (0.62–0.94)	No	No/0.002	71.0	7	3571/4344	53.96	5892	ACA	0.887	0.560	Weak	[56]
			CC + CT vs TT	Caucasian	0.63 (0.43–0.93)	No	NA/0.12	59.0	2	607/1009	52.73	1216	ACX	0.388	0.837	NA	[56]

NA: not available; No: significant publication bias was not found; present: significant publication bias was found; W vs M: wild allele vs mutant allele. ^†^Frequency of minor allele in controls. ^‡^The grade of C is assigned to protection from bias because the sensitivity analysis indicated that the significant summary OR can be substantially changed. ^§^The grade of A is assigned to protection from bias though the OR is less than 1.15, and the association is replicated by GWAS. ^¶^Frequency less than 1%, the amount of evidence is not applied considering an A grade is unlikely to obtain. ^※^Reported nonsignificantly in the diverse populations, but significant results were reported in the stratified analysis by ethnicity. ^*∗*^*P* value or *I*^2^ was calculated according to the original data provided in the article with Stata 12.0 since the article did not present these values. ^*∗∗*^*P* value of Egger's test/test for between-study heterogeneity.

**Table 2:** Statistically significant variants from GWASs.

Gene	Variants	Risk allele	Year	Ethnicity	OR (95% CI)	RAF^†^	*P* value^‡^	Cases/controls		Power OR of 1.5	FPRP values at prior probability of 0.01/0.001	Ref.
*CHRNA 3/5*	rs8034191	C	2009	Caucasian	NA	0.26^†^	1.48*E* − 10	1818	2567	—	—	[25]
*CHRNA 3/5*	rs1051730	T	2009	Caucasian	NA	0.27^†^	5.74*E* − 10	1818	2567	—	—	[25]
*FAM13A*	rs1903003	C	2010	Caucasian	NA	0.43^†^	9.47*E* − 11	4552	4582	—	—	[26]
*RAB4B-EGLN2*	rs7937	C	2012	Caucasian	0.73 (0.63–0.83)	0.44	2.88*E* − 09	3499	1922	0.917	0.000/0.002	[27]
*MIA-RAB4B*	rs2604894	A	2012	Caucasian	0.74 (0.65–0.84)	0.44	3.41*E* − 08	3499	1922	0.947	0.000/0.003	[27]
*FAM13A*	rs7671167	C	2012	Caucasian	0.73 (0.66–0.81)	0.49	2.22*E* − 09	3499	1922	0.956	0.000/0.000	[27]
*FAM13A*	rs1964516	C	2012	Caucasian	0.73 (0.66–0.81)	0.49	1.88*E* − 09	3499	1922	0.956	0.000/0.000	[27]
*CHRNA3*	rs12914385	T	2014	Diverse	1.28 (1.20–1.36)	0.31^†^	6.38*E* − 14	6633	5704	1.000	0.000/0.000	[28]
*FAM13A*	rs4416442	C	2014	Diverse	1.28 (1.20–1.36)	0.45^†^	1.12*E* − 14	6633	5704	1.000	0.000/0.000	[28]
*MMP12*	rs626750	G	2014	Diverse	1.36 (1.23–1.51)	0.80^†^	5.35*E* − 09	3497	5704	0.967	0.000/0.000	[28]
*HHIP*	rs13141641	T	2017	Diverse	1.22 (1.19–1.26)	0.59	9.10*E* − 41	24754	57684	1.000	0.000/0.000	[29]
*CHRNA5*	rs17486278	C	2017	Diverse	1.18 (1.15–1.22)	0.35	1.77*E* − 28	24754	57684	1.000	0.000/0.000	[29]
*HTR4*	rs7733088	G	2017	Diverse	1.18 (1.14–1.21)	0.60	5.33*E* − 26	24754	57684	1.000	0.000/0.000	[29]
*ADGRG6*	rs9399401	T	2017	Diverse	1.15 (1.12–1.19)	0.72	1.81*E* − 19	24754	57684	1.000	0.000/0.000	[29]
*THSD4*	rs1441358	G	2017	Diverse	1.13 (1.10–1.16)	0.33	8.22*E* − 16	24754	57684	1.000	0.000/0.000	[29]
*FAM13A*	rs6837671	G	2017	Diverse	1.12 (1.09–1.15)	0.41	7.48*E* − 15	24754	57684	1.000	0.000/0.000	[29]
*GSTCD*	rs11727735	A	2017	Diverse	1.26 (1.18–1.33)	0.94	3.84*E* − 14	24754	57684	1.000	0.000/0.000	[29]
*RIN3*	rs754388	C	2017	Diverse	1.15 (1.11–1.20)	0.82	4.96*E* − 14	24754	57684	1.000	0.000/0.000	[29]
*ADAM19*	rs113897301	A	2017	Diverse	1.16 (1.12–1.21)	0.17	1.58*E* − 13	24754	57684	1.000	0.000/0.000	[29]
*TET2*	rs2047409	A	2017	Diverse	1.12 (1.08–1.15)	0.62	2.46*E* − 13	24754	57684	1.000	0.000/0.000	[29]
*EEFSEC*	rs2955083	A	2017	Diverse	1.18 (1.13–1.24)	0.88	4.16*E* − 13	24754	57684	1.000	0.000/0.000	[29]
*CFDP1*	rs7186831	A	2017	Diverse	1.12 (1.08–1.16)	0.43	1.12*E* − 11	24754	57684	1.000	0.000/0.000	[29]
*TGFB2*	rs10429950	T	2017	Diverse	1.11 (1.07–1.14)	0.73	1.66*E* − 10	24754	57684	1.000	0.000/0.000	[29]
*AGER*	rs2070600	C	2017	Diverse	1.24 (1.16–1.32)	0.95	5.94*E* − 10	24754	57684	1.000	0.000/0.000	[29]
*ARMC2*	rs2806356	C	2017	Diverse	1.12 (1.08–1.16)	0.18	8.34*E* − 10	24754	57684	1.000	0.000/0.000	[29]
*PID1*	rs16825267	C	2017	Diverse	1.19 (1.12–1.25)	0.93	1.68*E* − 09	24754	57684	1.000	0.000/0.000	[29]
*DSP*	rs2076295	T	2017	Diverse	1.09 (1.06–1.12)	0.55	3.97*E* − 09	24754	57684	1.000	0.000/0.000	[29]
*MTCL1*	rs647097	C	2017	Diverse	1.10 (1.06–1.13)	0.27	6.14*E* − 09	24754	57684	1.000	0.000/0.000	[29]
*RARB*	rs1529672	C	2017	Diverse	1.11 (1.07–1.15)	0.83	2.47*E* − 08	24754	57684	1.000	0.003/0.026	[29]
*SFTPD*	rs721917	G	2017	Diverse	1.08 (1.05–1.11)	0.42	2.49*E* − 08	24754	57684	1.000	0.000/0.000	[29]
*CYP2A6*	rs12459249	C	2017	Diverse	1.10 (1.06–1.14)	0.66	3.42*E* − 08	24754	57684	1.000	0.000/0.000	[29]
*CCDC101*	rs17707300	C	2017	Diverse	1.10 (1.06–1.13)	0.37	NA	24754	57684	1.000	0.000/0.000	[29]

NA: not available. ^†^Risk allele frequency from the dbSNP. ^‡^The *P* values are all less than 5.00*E* − 08.

## Data Availability

All data generated or analyzed during this study are included in this published article and its supplementary information files.

## Supplementary Materials

Supplementary Materials Supplementary Table 1: statistically significant variants from the other two GWASs. Supplementary Table 2: statistically nonsignificant gene-variant COPD overall associations. Supplementary Table 3: conflicting gene-variant COPD overall associations.
