# Supplementary material for: Genetic Variants Associated with Chronic Obstructive Pulmonary Disease Risk: Cumulative Epidemiological Evidence from Meta-Analyses and Genome-Wide Association Studies
Source: Can Respir J. 2022 Jun 9;2022:3982335. doi: 10.1155/2022/3982335 (PMC9203202; doi:10.1155/2022/3982335)
Supplement: Supplementary file 1 [file 3982335.f1.docx]

Supplementary Table 1 Statistically significant variants from the other two GWASs

Gene	Variants	Risk Allele	Year	ethnicity	OR (95% CI)	RAF†	P value‡	Cases/control		Ref.
FAM13A	rs2869967	C	2014	Caucasian	1.38 (1.25-1.53)	0.49†	5.61E-10	1662	3520	[22]
FAM13A	rs2045517	T	2014	Caucasian	1.38 (1.25-1.53)	0.49†	5.63E-10	1662	3520	[22]
FAM13A	rs2904259	C	2014	Caucasian	1.35 (1.22-1.49)	0.47†	6.25E-09	1662	3520	[22]
EFCAB4A	rs34391416	A	2014	Caucasian	1.93 (1.53-2.45)	0.02†	4.99E-08	1662	3520	[22]
C1orf87	rs72673419	T	2019	Diverse	1.14 (1.09-1.19)	0.05	4.00E-09	35735	222076	[21]
DENND2D	rs629619	T	2019	Diverse	1.08 (1.06-1.11)	0.20	2.90E-10	35735	222076	[21]
DDX1	rs10929386	C	2019	Diverse	1.06 (1.04-1.08)	0.49	9.10E-09	35735	222076	[21]
SLMAP	rs62259026	C	2019	Diverse	1.07 (1.04-1.09)	0.75	2.40E-08	35735	222076	[21]
BTC	rs4585380	G	2019	Diverse	1.07 (1.05-1.09)	0.74	3.40E-09	35735	222076	[21]
FGF18	rs12519165	A	2019	Diverse	1.07 (1.05-1.09)	0.38	1.10E-09	35735	222076	[21]
CITED2	rs646695	C	2019	Diverse	1.08 (1.05-1.10)	0.24	4.60E-11	35735	222076	[21]
ITGB8	rs2040732	C	2019	Diverse	1.06 (1.04-1.08)	0.58	6.90E-09	35735	222076	[21]
STN1	rs1570221	A	2019	Diverse	1.06 (1.04-1.08)	0.35	2.20E-08	35735	222076	[21]
ARNTL	rs4757118	T	2019	Diverse	1.06 (1.04-1.08)	0.54	3.80E-09	35735	222076	[21]
SERP2	rs9525927	G	2019	Diverse	1.08 (1.05-1.10)	0.19	2.80E-09	35735	222076	[21]
DTWD1	rs72731149	G	2019	Diverse	1.12 (1.08-1.16)	0.91	8.30E-09	35735	222076	[21]
ADAMTSL3	rs10152300	G	2019	Diverse	1.08 (1.06-1.11)	0.23	4.20E-12	35735	222076	[21]
ASAP2	rs955277	T	2019	Diverse	1.07 (1.05-1.09)	0.61	1.90E-10	35735	222076	[21]
EML4	rs12466981	C	2019	Diverse	1.06 (1.04-1.09)	0.73	4.90E-08	35735	222076	[21]
VGLL4	rs2442776	G	2019	Diverse	1.09 (1.06-1.12)	0.15	2.00E-10	35735	222076	[21]
ADCY5	rs4093840	A	2019	Diverse	1.06 (1.04-1.08)	0.47	3.90E-10	35735	222076	[21]
HSPA4	rs62375246	A	2019	Diverse	1.06 (1.04-1.09)	0.26	2.20E-08	35735	222076	[21]
CCDC69	rs979453	G	2019	Diverse	1.06 (1.04-1.08)	0.34	1.40E-08	35735	222076	[21]
RREB1	rs1334576	A	2019	Diverse	1.06 (1.04-1.08)	0.42	1.20E-08	35735	222076	[21]
ID4	rs9350191	T	2019	Diverse	1.12 (1.09-1.15)	0.85	5.10E-14	35735	222076	[21]
IER3	rs2284174	C	2019	Diverse	1.12 (1.10-1.15)	0.22	2.10E-21	35735	222076	[21]
RFX6	rs674621	C	2019	Diverse	1.06 (1.04-1.08)	0.32	7.60E-09	35735	222076	[21]
MFHAS1	rs9329170	C	2019	Diverse	1.10 (1.07-1.13)	0.86	3.60E-10	35735	222076	[21]
COL15A1	rs10760580	G	2019	Diverse	1.07 (1.05-1.10)	0.71	1.20E-10	35735	222076	[21]
TEPP	rs8044657	G	2019	Diverse	1.11 (1.07-1.15)	0.90	1.10E-08	35735	222076	[21]
THRA	rs62065216	A	2019	Diverse	1.06 (1.04-1.08)	0.42	8.10E-09	35735	222076	[21]
TESK2	rs4660861	G	2019	Diverse	1.06 (1.04-1.08)	0.57	4.40E-08	35735	222076	[21]
ZBTB38	rs7650602	C	2019	Diverse	1.06 (1.04-1.08)	0.45	4.90E-08	35735	222076	[21]
TNPO1	rs34651	C	2019	Diverse	1.11 (1.07-1.15)	0.08	3.00E-08	35735	222076	[21]
AMZ1	rs798565	G	2019	Diverse	1.07 (1.04-1.09)	0.71	3.90E-09	35735	222076	[21]
RASEF	rs7866939	C	2019	Diverse	1.06 (1.04-1.08)	0.33	1.70E-08	35735	222076	[21]
MED13L	rs7958945	G	2019	Diverse	1.06 (1.04-1.09)	0.36	1.00E-09	35735	222076	[21]
DMWD	rs72626215	G	2019	Diverse	1.07 (1.05-1.10)	0.73	1.70E-09	35735	222076	[21]
SYN3	rs73158393	C	2019	Diverse	1.07 (1.05-1.09)	0.74	7.70E-09	35735	222076	[21]
MFAP2	rs9435731	A	2019	Diverse	1.06 (1.04-1.08)	0.51	6.60E-10	35735	222076	[21]
PABPC4	rs76841360	A	2019	Diverse	1.08 (1.05-1.10)	0.23	5.00E-10	35735	222076	[21]
TGFB2	rs3009947	C	2019	Diverse	1.06 (1.04-1.08)	0.50	4.00E-09	35735	222076	[21]
SLC30A10	rs11118406	T	2019	Diverse	1.08 (1.05-1.10)	0.28	4.10E-11	35735	222076	[21]
CHRM3	rs11579382	C	2019	Diverse	1.06 (1.04-1.08)	0.42	6.50E-09	35735	222076	[21]
NR4A2	rs72902175	T	2019	Diverse	1.09 (1.06-1.12)	0.13	1.60E-08	35735	222076	[21]
TNS1	rs2571445	A	2019	Diverse	1.07 (1.05-1.09)	0.39	3.40E-12	35735	222076	[21]
TWIST2	rs62191105	C	2019	Diverse	1.09 (1.07-1.12)	0.80	2.60E-12	35735	222076	[21]
RBMS3	rs13073544	C	2019	Diverse	1.06 (1.04-1.09)	0.28	2.00E-08	35735	222076	[21]
CACNA2D3	rs17759204	G	2019	Diverse	1.07 (1.05-1.10)	0.27	8.80E-11	35735	222076	[21]
MECOM	rs7642001	A	2019	Diverse	1.08 (1.06-1.10)	0.37	1.10E-14	35735	222076	[21]
FAM13A	rs7671261	A	2019	Diverse	1.09 (1.07-1.11)	0.55	1.40E-18	35735	222076	[21]
NPNT	rs34712979	A	2019	Diverse	1.18 (1.16-1.21)	0.25	3.00E-46	35735	222076	[21]
HHIP	rs13140176	A	2019	Diverse	1.18 (1.16-1.20)	0.61	4.10E-59	35735	222076	[21]
ITGA1	rs1551943	A	2019	Diverse	1.08 (1.05-1.10)	0.23	5.80E-10	35735	222076	[21]
SPATA9	rs153916	T	2019	Diverse	1.06 (1.04-1.08)	0.55	6.30E-10	35735	222076	[21]
HTR4	rs10037493	C	2019	Diverse	1.13 (1.10-1.15)	0.55	2.60E-33	35735	222076	[21]
ADAM19	rs10866659	G	2019	Diverse	1.09 (1.07-1.11)	0.35	1.20E-16	35735	222076	[21]
PRL	rs13198656	T	2019	Diverse	1.06 (1.04-1.08)	0.56	1.20E-09	35735	222076	[21]
ZKSCAN1	rs2897075	C	2019	Diverse	1.07 (1.04-1.09)	0.63	7.30E-10	35735	222076	[21]
GLIS3	rs10114763	T	2019	Diverse	1.07 (1.05-1.09)	0.42	8.70E-13	35735	222076	[21]
ELAVL2	rs156394	T	2019	Diverse	1.07 (1.05-1.09)	0.53	3.80E-12	35735	222076	[21]
ASTN2	rs803923	A	2019	Diverse	1.06 (1.04-1.08)	0.53	2.70E-08	35735	222076	[21]
CDC123	rs7068966	C	2019	Diverse	1.10 (1.08-1.12)	0.49	6.20E-23	35735	222076	[21]
LRMDA	rs2579762	C	2019	Diverse	1.06 (1.04-1.09)	0.47	2.60E-10	35735	222076	[21]
PRSS23	rs117261012	G	2019	Diverse	1.09 (1.06-1.12)	0.16	6.90E-10	35735	222076	[21]
CCDC91	rs11049386	T	2019	Diverse	1.06 (1.04-1.09)	0.71	2.70E-08	35735	222076	[21]
SNRPF	rs7307510	C	2019	Diverse	1.08 (1.05-1.10)	0.81	2.60E-09	35735	222076	[21]
RIN3	rs72699855	G	2019	Diverse	1.08 (1.05-1.11)	0.81	4.80E-09	35735	222076	[21]
CHRNA3	rs55676755	G	2019	Diverse	1.11 (1.09-1.14)	0.34	2.70E-26	35735	222076	[21]
TEKT5	rs56134392	C	2019	Diverse	1.06 (1.04-1.08)	0.35	4.50E-08	35735	222076	[21]
CFDP1	rs4888379	T	2019	Diverse	1.10 (1.08-1.12)	0.58	5.90E-21	35735	222076	[21]
EFCAB5	rs8080772	T	2019	Diverse	1.06 (1.04-1.08)	0.64	1.40E-08	35735	222076	[21]
RPL23	rs34727469	T	2019	Diverse	1.09 (1.06-1.12)	0.14	1.10E-08	35735	222076	[21]
SPPL2C	rs12373142	G	2019	Diverse	1.08 (1.05-1.10)	0.22	9.90E-10	35735	222076	[21]
SOX9	rs11655567	C	2019	Diverse	1.06 (1.04-1.08)	0.49	1.90E-09	35735	222076	[21]

Supplementary Table 2 Statistically non-significant gene-variant COPD overall associations

Gene	Variants	Year	Comparison	ethnicity	OR (95% CI)	Adjustment for smoking	Publication bias/ heterogeneity†	I^2(%)	No. of studies	Cases/control	Ref.
TNF-α	rs361525	2013	A vs G	Diverse	0.97 (0.69-1.37)	No	No/0.446	0.0	6	797/1257	[47]
TNF-α	rs1799964	2010	C vs T	Diverse	0.89 (0.67-1.17)	No	Na/Na	0.0	3	835‡	[9]
TNF-α	rs1799724	2010	C vs T	Diverse	1.04 (0.77-1.40)	No	Na/Na	0.0	3	803‡	[9]
TNF-α	rs1800610	2016	A vs G	Diverse	1.20 (0.96-1.50)	No	0.920/0.005	58.0	13	2459/2823	[58]
TGF-β1	rs1800469	2017	T vs C	Diverse	0.89 (0.77–1.02)	No	No/0.019	Na	9	2405/3338	[34]
TGF-β1	rs2241718	2017	T vs C	Diverse	0.95 (0.79-1.14)	No	No/0.428	Na	3	705/746	[34]
TGF-β1	rs2241712	2017	A vs G	Diverse	1.03 (0.89-1.20)	No	No/0.123	Na	4	807/906	[34]
TGF-β1	rs1982073	2017	C vs T	Diverse	0.87 (0.73-1.03)	No	No/<0.001	72.7	9	2365/3247	[34]
TGF-β1	rs6957	2017	A vs G	Diverse	1.14 (0.95-1.36)	No	No/0.084	Na	5	1732/2751	[34]
IL-1β	rs16944	2015	TT +CC vs CT	Diverse	0.89 (0.78-1.01)	No	0.756/0.32	12.0	13	1692/2009	[59]
IL-1β	rs1143634	2019	C vs T	Diverse	1.02 (0.72-1.44)	No	0.62/0.63	0.0	4	316/233	[35]
IL-1β	rs1143627	2009	CC+CT vs TT	Diverse	1.30 (0.99-1.71)	No	Na/0.1	Na	4	1777/4263	[8]
IL-4	rs2243250	2016	T vs C	Diverse	0.91 (0.72-1.14)	No	Na/0.06	47.0	8	1400/1073	[60]
IL-4	rs2070874	2010	C vs T	Diverse	1.05 (0.79-1.38)	No	Na/Na	0.0	4	596‡	[9]
IL-6	rs1800797	2016	A vs G	Caucasian	1.12 (0.97-1.28)	No	Na/0.128	47.3	4	1126/986	[61]
IL-13	rs20541	2017	A vs G	Diverse	1.12 (0.96-1.30)	No	Na/0.389	Na	7	1418/763	[36]
IL-27	rs181206	2016	C vs T	Asian	0.73 (0.37-1.44)	No	Na/Na	Na	1	120/100	[37]
MMP-1	rs1799750	2013	1G vs 2G	Diverse	0.99 (0.89-1.10)	No	0.980/ 0.01	53.0	12	3207/4394	[62]
MMP-3	rs3025058	2016	6A vs 5A	Diverse	0.95 (0.77-1.17)	No	No/0.36	6.7	4	522/520	[63]
MMP-12	rs2276109	2013	A vs G	Diverse	0.98 (0.80-1.20)	No	0.066/ 0.11	44.0	6	2127/3054	[62]
SOD1	rs2234694	2018	CA vs AA	Diverse	1.31 (0.88-1.95)	No	No/0.388	0.7	4	518/787	[64]
SOD2	rs4880	2018	TC vs TT	Diverse	0.92 (0.72-1.18)	No	No/0.376	6.9	7	561/571	[64]
SOD3	rs1799895	2018	CA vs AA	Diverse	0.98 (0.63-1.53)	No	No/0.002	68.8	8	5441/95336	[64]
ADRB2	rs1042713	2014	G vs A	Diverse	1.07 (0.86-1.34)	No	0.211/0.001	Na	11	1128/1182	[65]
ADRB2	rs1042714	2018	G vs C	Diverse	1.18 (0.88-1.59)	No	0.15/0.0005	Na	8	1156/1026	[55]
ADRB2	rs1800888	2017	T vs C	Diverse	2.57 (0.54-12.36)	No	Na/Na	Na	2	Na	[66]
SERPINA3	Ala9Thr	2010	A vs G	Diverse	0.80 (0.62-1.03)	No	Na/Na	45.0	5	1338‡	[9]
SERPINE2	rs3795879	2015	G vs A	Diverse	1.23 (0.97-1.32)	No	0.396/0.043	59.3	5	3034/3068	[67]
CHRNA5	rs578776	2010	AA vs GG	Caucasian	0.93 (Na)	Yes	Na/Na	Na	3	2609/3542	[68]
CHRNA5	rs588765	2010	CC vs TT	Caucasian	0.92 (Na)	Yes	Na/Na	Na	3	2607/3548	[68]
CHRNA5	rs12914008	2010	AA vs GG	Caucasian	0.86 (Na)	Yes	Na/Na	Na	3	2609 3549	[68]
ADAM33	rs2787094	2014	G vs C	Diverse	0.95 (0.76-1.19)	No	0.945/Na	79.0	7	1738/3643	[42]
ADAM33	rs2280089	2014	A vs G	Asian	0.88 (0.72-1.07)	No	0.097/Na	47.6	3	767/773	[42]
ADAM33	rs2280090	2014	A vs G	Diverse	0.66 (0.38-1.13)	No	0.658/0.000	92.9	5	1062/3032	[42]
ADAM33	rs528557	2014	G vs C	Diverse	0.97 (0.74-1.27)	No	0.420/Na	85.5	10	1774/3789	[42]
SP-B	Common Polymorph	2013	W vs M	Diverse	1.13 (0.73-1.74)	No	> 0.05/0.002	79.9	3	493/616	[44]
SP-D	Common Polymorph	2013	W vs M	Diverse	1.09 (0.85-1.40)	No	> 0.05/< 0.001	87.0	2	504/578	[44]
SP-D	rs2243639	2019	G vs A	Diverse	0.99 (0.69-1.43)	No	0.610/0.080	55.6	4	483/349	[45]
VDBP	GC-2	2019	2 vs Non 2	Diverse	0.92 (0.76-1.11)	No	No/0.007	56.0	13	1540/1725	[69]
GSTP1	rs1695	2009	AA+GA vs GG	Diverse	0.89 (0.73-1.10)	No	Na/0.013	Na	12	1507/3083	[8]
ACE	I/D	2018	D vs I	Diverse	1.14 (0.91-1.41)	No	No/0.000	66.2	15	1279/1356	[54]
LTA	rs909253	2010	A vs G	Diverse	1.00 (0.78-1.28)	No	Na/Na	0.0	4	798‡	[9]
TIMP2	rs2277698	2010	A vs G	Diverse	0.59 (0.23-1.48)	No	Na/Na	89.0	3	1051‡	[9]
COX2	rs689466	2014	A vs G	Asian	1.26 (0.97-1.62)	No	>0.05/0.653	0.0	2	230/273	[39]

Na: not available; No: significant publication bias was not found; W vs M: wild allele vs mutant allele

† *P* value of the Egger's test/test for between-study heterogeneity

‡ Total sample size

Supplementary Table 3 Conflicting gene-variant COPD overall associations

Gene	Variants	Year	Comparison	ethnicity	OR (95% CI)	Adjustment for smoking	Publication bias/ heterogeneity	I^2(%)	No. of studies	Cases/control	Ref.
TNF-α	rs1800629	2007	A vs G	Asian	2.62 (2.00-3.43)	No	Na/0.53	Na	10	666/898	[70]
			A vs G	Asian	2.18 (1.41-3.37)	Yes	Na/0.13	Na	5	358/321	[70]
TNF-α	rs1800629	2009	Dominant model	Diverse	1.06 (0.95-1.17)	No	Na/0.27	Na	21	3552/3639	[8]
			Dominant model	Asian	2.01 (1.21-3.31)	No	Na/0.15	Na	5	283/385	[8]
TNF-α	rs1800629	2010	A vs G	Diverse	1.19 (1.01-1.40)	No	Na/Na	34.0	27	2515[†]	[9]
TNF-α	rs1800629	2010	A vs G	Diverse	1.33 (1.17-1.51)	No	0.368/0.000	Na	21	2380/3738	[71]
			A vs G	Asian	2.33 (1.88-2.88)	No	No/0.477	Na	14	1092/1794	[71]
			A vs G	Asian	2.39 (1.94-2.75)	Yes	No/0.689	Na	6	760/1009	[71]
TNF-α	rs1800629	2010	Additive model	Diverse	1.48 (1.24-1.77)	No	0.017/ 0.000	74.0	36	4975/6518	[72]
			Additive model	Asian	2.36 (1.84-3.02)	No	Na/0.009	48.8	19	1585/2842	[72]
			Additive model	Diverse	1.35 (1.06-1.73)	Yes	0.262/ 0.000	64.9	21	3018/2749	[72]
			Additive model	Asian	1.72 (1.14-2.61)	Yes	No/0.029	48.6	12	844/752	[72]
TNF-α	rs1800629	2016	A vs G	Diverse	1.56 (1.29-1.89)	No	No/0.000	70.8	38	3951/5110	[46]
			A vs G	Asian	2.40 (1.98-2.90)	No	No/0.098	29.8	22	2067/2167	[46]
			A vs G	Diverse	1.13 (0.95-1.35)	Yes	No/0.089	34.9	15	1500/1239	[46]
			A vs G	Asian	1.26 (0.69-2.30)	Yes	No/0.063	52.2	6	570/616	[46]
TNF-α	rs1800629	2018	A vs G	Asian	2.25 (1.84-2.76)	No	No/0.96	0.0	9	657/568	[73]
TNF-α	rs1800629	2016	A vs G	Asian	2.54 (2.05-3.14)	No	>0.05/0.483	0.0	11	953/1079	[74]
TNF-α	rs1800629	2019	A vs G	Diverse	1.39 (0.98-1.97)	No	0.360/< 0.00	93.2	46	5402/ 7247	[96]
TNF-α	rs1800629	2019	A vs G	Asian	2.45 (1.93-3.11)	No	No/0.013	47.6	18	1817/2056	[97]
TNF-α	rs1800630	2009	Dominant model	Diverse	1.10 (0.92-1.33)	No	Na/0.26	Na	6	1692/1596	[8]
TNF-α	rs1800630	2010	A vs G	Diverse	1.26 (0.96-1.66)	No	Na/Na	0.0	6	442[†]	[9]
TNF-α	rs1800630	2015	A vs G	Diverse	1.39 (1.01-1.90)	No	No/0.004	63.1	10	1113/1439	[75]
			A vs G	Asian	1.58 (1.04-2.42)	No	/0.024	61.3	6	496/834	[75]
TNF-α	rs1800630	2017	A vs G	Asian	1.55 (1.02-2.33)	No	No/0.057	53.5	6	468/724	[76]
TNF-α	rs1800630	2016	A vs G	Diverse	1.20 (0.96-1.50)	No	0.920/0.005	58.0	13	2459/2823	[58]
TNF-α	rs1800630	2010	A vs C	Diverse	0.91 (0.67-1.24)	No	Na/Na	0.0	3	823[†]	[9]
TNF-α	rs1800630	2013	A vs C	Diverse	0.76 (0.60-0.97)	No	No/0.158	42.3	4	606/622	[47]
			A vs C	Asian	0.56 (0.37-0.85)	No	Na/0.640	0.0	2	202/203	[47]
GSTT1	NullPresent	2008	Null vs. Present	Diverse	1.06 (0.79-1.42)	No	No/ 0.03	53.8	8	983/1158	[77]
GSTT1	NullPresent	2009	Recessive model	Diverse	1.00 (0.82-1.22)	No	Na/0.19	Na	7	929/1021	[8]
GSTT1	NullPresent	2010	Null vs. Present	Diverse	0.92 (0.73-1.15)	No	Na/Na	0.0	5	1408[†]	[9]
GSTT1	NullPresent	2011	Null vs. Present	Diverse	1.05 (0.87-1.26)	No	No/0.16	32.6	9	1053/1079	[78]
GSTT1	NullPresent	2018	Null vs. Present	Diverse	1.28 (1.09-1.50)	No	No/0.001	51.0	29	3273/3578	[32]
			Null vs. Present	Asian	1.30 (1.05-1.61)	No	No/0.002	57.0	18	2025/2253	[32]
TGF-β1	rs2241712	2009	Dominant model	Diverse	0.73 (0.57-0.94)	No	Na/0.83	Na	3	476/562	[8]
TGF-β1	rs2241712	2017	Allele Model	Diverse	1.03 (0.89-1.20)	No	No/0.123	Na	4	807/906	[34]
TGF-β1	rs1982073	2009	Dominant model	Diverse	0.70 (0.58-0.84)	No	Na/0.7	Na	5	3552/3639	[8]
TGF-β1	rs1982073	2011	TT+TC vs CC	Diverse	1.06 (0.70-1.60)	No	>0.05/<0.0001	77.0	8	1267/2389	[79]
			TT+TC vs CC	Diverse	1.18 (0.75-1.86)	Yes	>0.05/0.02	68.0		678/894	[79]
TGF-β1	rs1982073	2017	Allele Model	Diverse	0.87 (0.73-1.03)	No	No/<0.001	72.7	9	2365/3247	[34]
			Allele Model	Caucasian	0.79 (0.64-0.99)	No	No/0.006	75.7	4	1656/2564	[34]
TGF-β1	rs6957	2009	Dominant model	Diverse	1.18 (1.13-1.93)	No	Na/0.29	Na	3	558/1668	[8]
TGF-β1	rs6957	2017	Allele Model	Diverse	1.14 (0.95-1.36)	No	No/0.084	Na	5	1732/2751	[34]
IL1β	rs1143627	2009	Dominant model	Diverse	1.30 (0.99-1.71)	No	Na/0.1	Na	4	1777/4263	[8]
IL1β	rs1143627	2010	C vs T	Diverse	0.95 (0.62-1.46)	No	Na/Na	54.0	4	533[†]	[9]
IL1β	rs1143627	2013	Additive model	Diverse	0.96 (0.74-1.24)	No	0.713/0.094	49.6	5	620/702	[80]
			Additive model	Caucasian	0.62 (0.40-0.97)	No	Na/Na	Na	1	106/72	[80]
IL1β	rs1143627	2014	CC+TT vs TC	Diverse	0.77 (0.63-0.94)	No	0.138/0.59	0.0	6	764/879	[81]
			CC+TT vs TC	Asian	0.75 (0.61-0.93)	No	No/0.50	0.0	5	639/808	[81]
IL1β	rs1143627	2014	Dominant model	Diverse	1.25 (0.79-1.96)	No	Na/0.101	Na	4	534/632	[82]
			Dominant model	Asian	1.55 (1.14-2.11)	No	Na/0.842	Na	3	429/561	[82]
IL1β	rs1143627	2019	C vs T	Diverse	1.11 (0.94-1.31)	No	0.76/0.89	0.0	5	474/816	[35]
IL1β	rs1143627	2013	C vs T	Asian	0.99 (0.86-1.15)	No	0.784/0.13	43.0	3	503/727	[83]
MMP-9	rs3918242	2009	Dominant model	Diverse	1.45 (0.84-2.50)	No	Na/0.0063	Na	5	668/822	[8]
MMP-9	rs3918242	2010	C vs T	Diverse	0.69 (0.44-1.09)	No	Na/Na	42.0	4	850[†]	[9]
MMP-9	rs3918242	2013	Additive model	Diverse	1.35 (1.00-1.82)	No	0.570/Na	Na	11	Na	[84]
			Additive model	Diverse	1.71 (1.42-2.07)	Yes	No/0.10	43.0	7	846/919	[84]
MMP-9	rs3918242	2013	C vs T	Diverse	0.83 (0.62-1.12)	No	0.953/<0.0001	79.0	12	2009/2233	[62]
MMP-9	rs3918242	2016	T vs C	Diverse	1.36 (1.16-1.61)	No	No/0.261	21.2	8	1067/1091	[63]
MMP-9	rs3918242	2017	T vs C	Diverse	1.32 (1.02-1.71)	No	0.12/<0.001	77.0	13	2296/2539	[38]
			CT+TT vs CC	Asian	1.80 (1.04-3.11)	No	No/<0.001	85.0	8	1101/1147	[38]
EPHX1	rs1051740	2008	His/His vs Tyr/Tyr	Diverse	1.59 (1.14-2.21)	No	No/0.009	62.6	16	1847/2455	[85]
			His/His vs Tyr/Tyr	Asian	1.57 (1.24-1.98)	No	No/0.15	34.0	9	1035/1106	[85]
EPHX1	rs1051740	2009	Dominant model	Diverse	1.02 (0.91-1.14)	No	Na/0.47	Na	12	2438/3975	[8]
EPHX1	rs1051740	2010	C vs T	Diverse	1.11 (0.95-1.30)	No	Na/Na	65.0	15	4687[†]	[9]
EPHX1	rs1051740	2011	CC vs TT	Diverse	1.38 (1.09-1.74)	No	Na/Na	66.0	19	7404/42970	[86]
			CC vs TT	Caucasian	1.40 (1.02-1.94)	No	Na/Na	70.0		6379/41816	[86]
EPHX1	rs1051740	2012	CC vs TT	Diverse	1.33 (1.06- 1.69)	No	0.158/<0.0001	73.0	25	4808/24888	[48]
			CC vs TT	Caucasian	1.61 (1.12- 2.31)	No	No/<0.0001	81.0	12	3930/23907	[48]
EPHX1	rs1051740	2016	C vs T	Caucasian	1.12 (1.00-1.25)	No	No/0.01	58.3	9	6470/40577	[87]
			C vs T	Asian	1.04 (0.86-1.25)	No	No/0.01	57.8	10	1228/1358	[87]
EPHX1	rs2234922	2008	Arg/Arg vs His/His	Diverse	0.90 (0.65-1.24)	No	No/0.53	0.0	15	343/2625	[85]
EPHX1	rs2234922	2009	Dominant model	Diverse	0.91 (0.83-1.01)	No	Na/0.059	Na	18	3047/4821	[8]

SOD3	rs1799895	2009	Dominant model	Caucasian	0.63(0.25-1.60)	No	Na/0.0089	Na	3	1363/8979	[8]
SOD3	rs1799895	2010	C vs G	Diverse	1.97(1.24-3.13)	No	Na/Na	0.0	3	9196[†]	[9]
SOD3	rs1799895	2018	CA vs AA	Diverse	0.98(0.63-1.53)	No	No/0.002	68.8	8	5441/95336	[64]
IL-13	rs1800925	2010	C vs T	Diverse	0.73(0.35-1.56)	No	Na/Na	83.0	3	536[†]	[9]
IL-13	rs1800925	2013	TT vs CC	Diverse	2.02(1.10-3.72)	No	0.185/0.09	43.0	8	943/625	[88]
			TT vs CC	Arabians	3.05(1.08-8.60)	No	Na/0.82	0.0	2	116/67	[88]
IL-13	rs1800925	2017	Allelic Model	Diverse	1.57(1.21-2.04)	No	Na/0.015	57.9	9	1477/891	[36]
			Allelic Model	Asian	1.88(1.23-2.87)	No	Na/0.016	67.1	5	555/544	[36]
			Allelic Model	Caucasian	1.30(1.01-1.67)	No	Na/0.314	15.6	4	922/347	[36]
IL-13	rs1800925	2019	C vs T	Diverse	0.70(0.59-0.84)	No	0.28/0.12	47.1	5	1035/502	[35]
IL-6	rs1800796	2016	C vs G	Diverse	0.56(0.31-1.03)	No	0.722/0.037	64.6	5	667/650	[61]
			C vs G	Caucasian	0.54(0.35-0.82)	No	Na/0.593	0.0	2	191/371	[61]
IL-6	rs1800796	2019	CC + GC vs GG	Diverse	2.23(1.46-3.40)	No	0.05/0.38	19.3	3	574/862	[35]
ADAM33	rs2280091	2014	GG vs AA	Diverse	2.12(0.87-5.19)	No	No/0.005	71.0	6	1263/2551	[89]
ADAM33	rs2280091	2014	G vs A	Diverse	1.33(0.88-1.99)	No	No/Na	91.0	7	2100/3526	[41]
			G vs A	Asian	2.23(1.38-3.60)	No	Na/Na	77.0	3	915/912	[41]
ADAM33	rs2280091	2014	Recessive model	Asian	3.50 (2.11-5.81)	No	0.750/0.16	46.0	3	915/912	[90]
ADAM33	rs2280091	2014	Recessive model	Diverse	1.76 (1.27-2.43)	No	0.483/0.348	10.5	8	2112/3952	[42]
			Allelic Model	Asian	2.03 (1.40–2.94)	No	0.857/0.006	76.0	4	1130/1135	[42]
ADAM33	rs2280091	2013	G vs A	Diverse	2.05(0.68-6.21)	No	0.506/0.000	97.7	5	1593/1737	[91]
ADAM33	rs528557	2014	CC vs GG	Diverse	0.77(0.63-0.94)	No	Na/Na	Na	9	1762/3363	[89]
ADAM33	rs528557	2014	C vs G	Diverse	1.03(0.79-1.33)	No	No/Na	82.0	8	1612/3210	[41]
ADAM33	rs528557	2014	Recessive model	Asian	0.86(0.69-1.08)	No	0.309/0.12	46.0	5	838/831	[90]
ADAM33	rs528557	2014	Allelic Model	Diverse	0.97(0.74-1.27)	No	0.420/Na	85.5	10	1774/3789	[42]
ADAM33	rs511898	2014	A vs G	Diverse	1.09(0.96-1.25)	No	No/Na	44.0	7	1588/3175	[41]
ADAM33	rs511898	2014	Allelic Model	Diverse	1.18 (1.02-1.38)	No	0.339/0.017	59.0	8	1600/3602	[42]
			Additive Model	Asian	1.32 (1.00–1.75)	No	0.428/0.167	40.8	4	870/863	[42]
ADAM33	rs511898	2013	A vs G	Diverse	1.13(0.76-1.68)	No	0.527/0.000	82.6	5	1092/1103	[91]
ADAM33	rs511898	2019	A vs G	Diverse	1.16(1.04-1.30)	No	0.726/0.048	44.5	12	2454/4481	[51]
ADAM33	rs612709	2014	A vs G	Diverse	1.46(1.14-1.87)	No	No/Na	66.0	5	1329/2933	[41]
			AA+AG vs GG	Asian	2.75(1.55-4.90)	No	Na/Na	50.0	2	552/540	[41]
			A vs G	Caucasian	1.38(1.16-1.64)	No	Na/Na	48.0	3	777/2393	[41]
			A vs G	Diverse	1.53(1.07-2.18)	Yes	Na/Na	69.0	3	836/849	[41]
ADAM33	rs612709	2014	Allelic Model	Diverse	0.60(0.52-0.68)	No	0.070/0.107	44.7	6	1341/3339	[42]
			Allelic Model	Asian	0.61(0.42-0.89)	No	0.256/0.021	74.1	3	767/763	[42]
			Allelic Model	Caucasian	0.64(0.53-0.77)	No	0.324/0.798	0.0	3	574/2596	[42]
ADAM33	rs612709	2013	A vs G	Diverse	0.57(0.32-1.01)	No	0.047/0.000	87.4	3	839/851	[91]
SP-D	Common Polymorph	2013	W vs M	Diverse	1.09(0.85-1.40)	No	> 0.05/<0.001	87.0	2	504/578	[44]
SP-D	rs721917	2019	T vs C	Asian	1.34(1.07-1.67)	No	No/0.73	0.0	3	334/328	[92]
SP-D	rs2243639	2019	G vs A	Diverse	0.99(0.69-1.43)	No	0.610/0.080	55.6	4	483/349	[45]
SP-D	rs721917	2019	T vs C	Diverse	1.39(1.18-1.65)	No	0.375/0.901	0.0	5	561/550	[45]
			T vs C	Asian	1.44(1.20-1.73)	No	0.457/0.978	0.0	4	499/435	[45]
VDBP	1F	2015	GC-1F vs GC-1S	Diverse	1.44(1.14-1.83)	No	N0/0.236	26.0	6	531/1181	[93]
			1F-1F vs 1S-1S	Diverse	2.64(1.29–5.39)	No	N0/0.750	0.0	6	531/1181	[93]
VDBP	1F	2015	1F-1F vs non 1F-1F	Diverse	2.05(1.23-3.43)	No	>0.05/0.004	65.0	9	913/1223	[94]
VDBP	1F	2015	1F vs 1S	Diverse	1.15(0.89-1.50)	No	0.942/0.08	47.0	7	734/741	[95]
			1F vs 1S	Asian	1.52(1.16-2.00)	No	No/0.82	0.0	4	335/322	[95]
			1F vs 2	Diverse	1.24(0.81-1.88)	No	0.557/0.0002	77.0	7	734/741	[95]
			1F vs 2	Asian	1.87(1.42-2.44)	No	No/0.39	0.0	4	335/322	[95]
			1F vs 2	Caucasian	0.73(0.54-0.98)	No	No/0.40	0.0	3	399/419	[95]
VDBP	1F	2015	1F vs 1S	Diverse	1.47(1.22-1.77)	No	No/0.447	0.0	8	809/1407	[49]
			1F vs 1S	Asian	1.53(1.22-1.92)	No	0.133/0.935	0.0	5	510/460	[49]
VDBP	1F	2015	1F vs Non 1F	Diverse	1.27(1.04-1.54)	No	No/0.005	60.0	11	1410/1595	[50]
			1F vs Non 1F	Asian	1.35(1.04-1.75)	No	No/0.007	66.0	7	785/719	[50]
VDBP	1F	2015	1F vs 2	Diverse	1.77(1.20-2.62)	No	No/0.001	72.2	8	809/1407	[49]
VDBP	1F	2015	1F vs 2	Asian	1.82(1.16-2.85)	No	0.183/0.003	74.7	5	510/460	[49]
VDBP	1F	2019	1F vs Non 1F	Diverse	1.29(1.09-1.55)	No	No/0.02	52.0	13	1540/1725	[69]
				Asian	1.45(1.24-1.68)	No	No/0.59	0.0	8	784/743	[69]
VDBP	2	2015	GC-2 vs GC-1S	Diverse	0.83(0.69–0.996)	No	N0/0.207	31.0	6	531/1181	[93]
VDBP	2	2015	2-2 vs non 2-2	Diverse	0.61(0.31-1.21)	No	>0.05/0.000	71.5	12	1190/1747	[94]
			2-2 vs non 2-2	Asian	0.21(0.11-0.43)	No	>0.05/0.387	3.5	5	410/391	[94]
VDBP	2	2015	2 vs Non 2	Diverse	0.90(0.72-1.11)	No	No/0.0003	68.0	12	1485/1639	[50]
VDBP	2	2019	2 vs Non 2	Diverse	0.92(0.76-1.11)	No	No/0.007	56.0	13	1540/1725	[69]
VDBP	1S	2015	1S vs 2	Diverse	1.15(0.88-1.50)	No	No/0.017	58.9	8	809/1407	[49]
VDBP	1S	2019	1S vs Non 1S	Diverse	0.96(0.74-1.25)	No	No/<0.00001	82.0	13	1540/1699	[69]
VDBP	1S	2015	1S vs Non 1S	Diverse	0.86(0.77-0.96)	No	No/0.12	35.0	11	1410/1595	[50]
			1S vs Non 1S	Asian	0.76(0.64-0.89)	No	No/0.23	26.0	7	785/719	[50]
HHIP	rs13118928	2010	GG vs AA	Diverse	0.77 (0.70–0.84)	No	Na/0.444	0.0	3	Na	[98]
HHIP	rs13118928	2019	G vs A	Diverse	1.14(1.08- 1.20)	No	No/0.930	0.0	7	5157/9768	[57]

Na: not available; No: significant publication bias was not found; W vs M: wild allele vs mutant allele

[1] P value of the Egger's test/test for between-study heterogeneity

[†]Total sample size
